# DNA fusion-gene vaccination in patients with prostate cancer induces high-frequency CD8^+^ T-cell responses and increases PSA doubling time

**DOI:** 10.1007/s00262-012-1270-0

**Published:** 2012-05-22

**Authors:** Lindsey Chudley, Katy McCann, Ann Mander, Torunn Tjelle, Juan Campos-Perez, Rosemary Godeseth, Antonia Creak, James Dobbyn, Bernadette Johnson, Paul Bass, Catherine Heath, Paul Kerr, Iacob Mathiesen, David Dearnaley, Freda Stevenson, Christian Ottensmeier

**Affiliations:** 1 Experimental Cancer Medicine Centre, Cancer Sciences Unit, Faculty of Medicine, University of Southampton, Tremona Road, Southampton, SO16 6YD UK; 2 Cancer Sciences Unit, Faculty of Medicine, University of Southampton, Tremona Road, Southampton, SO16 6YD UK; 3 Inovio Pharmaceuticals Inc., 1787 Sentry Parkway West, Building 18, Suite 400, Blue Bell, PA 19422 USA; 4 Royal Marsden Hospital and Institute of Cancer Research, Downs Road, Sutton, Surrey SM2 5PT UK; 5 University Hospitals Southampton NHS Foundation Trust, Southampton General Hospital, Tremona Road, Southampton, SO16 6YD UK; 6 Royal Devon and Exeter NHS Foundation Trust, Barrack Road, Exeter, Devon EX2 5DW UK

**Keywords:** Immunotherapy, Prostate cancer, DNA vaccine, Electroporation, CD8^+^ T cells

## Abstract

We report on the immunogenicity and clinical effects in a phase I/II dose escalation trial of a DNA fusion vaccine in patients with prostate cancer. The vaccine encodes a domain (DOM) from fragment C of tetanus toxin linked to an HLA-A2-binding epitope from prostate-specific membrane antigen (PSMA), PSMA_27–35_. We evaluated the effect of intramuscular vaccination without or with electroporation (EP) on vaccine potency. Thirty-two HLA-A2^+^ patients were vaccinated and monitored for immune and clinical responses for a follow-up period of 72 weeks. At week 24, cross-over to the immunologically more effective delivery modality was permitted; this was shown to be with EP based on early antibody data, and subsequently, 13/15 patients crossed to the +EP arm. Thirty-two HLA-A2^−^ control patients were assessed for time to next treatment and overall survival. Vaccination was safe and well tolerated. The vaccine induced DOM-specific CD4^+^ and PSMA_27_-specific CD8^+^ T cells, which were detectable at significant levels above baseline at the end of the study (*p* = 0.0223 and *p* = 0.00248, respectively). Of 30 patients, 29 had a measurable CD4^+^ T-cell response and PSMA_27_-specific CD8^+^ T cells were detected in 16/30 patients, with or without EP. At week 24, before cross-over, both delivery methods led to increased CD4^+^ and CD8^+^ vaccine-specific T cells with a trend to a greater effect with EP. PSA doubling time increased significantly from 11.97 months pre-treatment to 16.82 months over the 72-week follow-up (*p* = 0.0417), with no clear differential effect of EP. The high frequency of immunological responses to DOM-PSMA_27_ vaccination and the clinical effects are sufficiently promising to warrant further, randomized testing.

**Electronic supplementary material:**

The online version of this article (doi:10.1007/s00262-012-1270-0) contains supplementary material, which is available to authorized users.

## Introduction

Activating immunity against cancer in patients has been a difficult goal [1] but randomized studies are now showing encouraging results in solid tumors [2, 3], including prostate cancer [4]. Prostate cancer immunotherapy is attractive at early biochemical detection of recurrence since rising prostate-specific antigen (PSA), even without radiologically measurable disease, identifies patients at risk who have very small volume disease [5]. Vaccine targets, like Muc-1 [6], PSA [7, 8], prostatic acid phosphatase (PAP) [9] or prostate-specific membrane antigen (PSMA) [10–12], have been identified as promising targets [13]. A randomized phase III trial showed that prostate-associated antigens can be effectively targeted by vaccination [4]. The improved median survival of 4.1 months in late-stage disease was not mirrored by PSA changes [4], an observation also made in other immunotherapy studies [14]. Although Sipuleucel-T sets a treatment paradigm, producing a new patient-specific vaccine is a technical, financial and logistical challenge. Overall benefit remains small, indicating an unmet clinical need for better, ideally non-toxic, treatments to improve outcomes [13].

Vaccination against cancer using exogenous peptide has been tested widely and may confer clinical effect in some settings [15–17]. However, CD8^+^ T-cell responses following vaccination using exogenous short peptides appear transient [18] possibly due to the lack of T-cell help. Viral vector–based vaccines may overcome this problem and have shown promise in metastatic disease [8, 19] with effects also on PSA doubling time (PSA-DT) at biochemical failure [6]. However, viral vectors will either face pre-existing immunity or induce it on repeat injections. DNA vaccines avoid this problem and offer a novel delivery vehicle for the induction of peptide-specific responses.

We have designed DNA fusion-gene vaccines able to deliver tumor-derived peptides, together with microbial genes, to generate high levels of T-cell help [20]. Our platform design includes a strongly immunogenic helper domain (DOM), derived from fragment C (FrC) of tetanus toxin, linked to a tumor-epitope sequence of choice [20]. In pre-clinical models, DOM-epitope vaccines induce durable tolerance-breaking epitope-specific CD8^+^ T-cell immunity, able to suppress a range of tumors [20].

In mice expressing the HLA-A0201* transgene, the DOM-epitope vaccine design incorporating an epitope from PSMA (PSMA_27_ VLAGGFFLL) [20, 21] induced high levels of specific CD8^+^ T cells able to kill tumor cells [22]. We have now vaccinated patients with biochemically recurrent prostate cancer and, to optimize human translation, also evaluated delivery with electroporation (EP). EP has been reported to increase the potency of DNA vaccines by increasing antigen levels and stimulating local inflammation [23], and its use is rapidly expanding in both infectious diseases and cancer vaccination. We found that this approach was safe, well tolerated and significantly increased antibody induction [24].

We report here the effect of our DOM-epitope vaccine on T-cell immunity and clinical outcome. The vaccine reproducibly induces T-cell immunity to PSMA_27_ and significantly increases PSA-DT, and in spite of the small sample size, we identified a trend to increased time to next treatment compared to a control group of unvaccinated HLA-A2^−^ patients. Taken together, these data support further randomized testing of the vaccine.

## Patients and methods

### Patient population and regulatory information

Patients with biochemically recurrent prostate cancer, rising PSA (<50 ng/mL, PSA-DT >3 months) without radiological evidence of distant disease by CT scan, bone scan and/or MRI were eligible. Pelvic nodal enlargement up to 2 cm was allowed. Tumor PSMA expression was confirmed immunohistochemically at Southampton Cellular Pathology Laboratory. Other inclusion and exclusion criteria have been reported previously [24]. Patients were HLA-typed in NHS laboratories. The vaccine encodes an HLA-A2-restricted epitope; only HLA-A2^+^ patients were vaccinated. HLA-A2^−^ patients who fulfilled all other entry criteria formed the control group and were followed for the evaluation of time to next treatment and survival only.

Regulatory approval for the study was given by the UK Medicines and Healthcare Regulatory Authority (MHRA), the Gene Therapy Advisory Committee and local Research Ethics committees. The study was registered in the database of gene therapy trials in the UK. All patients gave written informed consent to participate in the study between March 2005 and February 2008 at the University Hospitals Southampton and the Royal Marsden Hospital.

### Study design

**Fig. 1:**
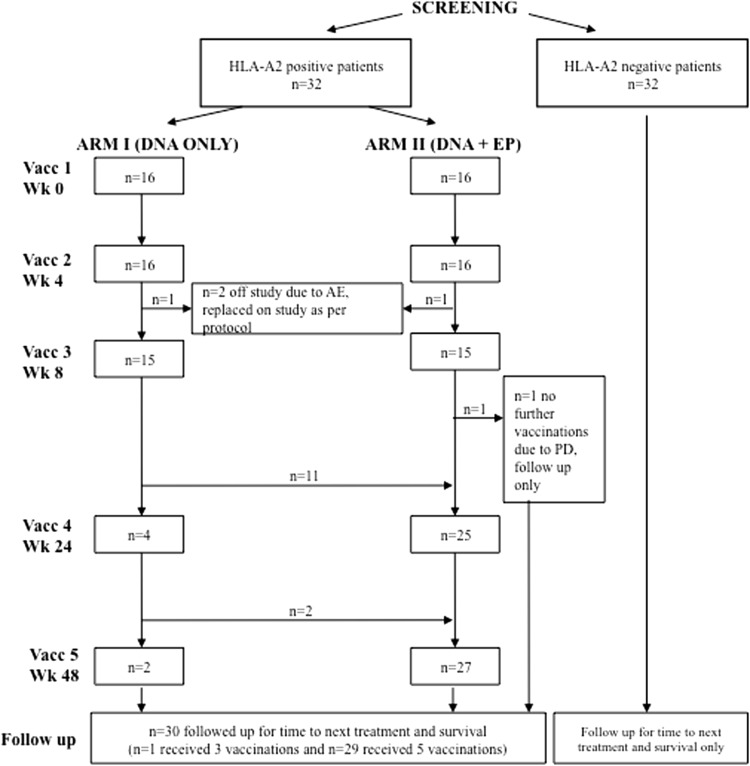
CONSORT diagram

The study was a phase I/II, open-label, non-randomized, two-center, dose escalation study. DOM-PSMA_27_ vaccine [20, 22, 24] was injected into the thigh muscle 5 times at 0, 4, 8, 24 and 48 weeks. HLA-A2^+^ patients were recruited to two study arms (Fig. 1). In arm I, patients received DNA intramuscularly (i.m.), and in arm II, vaccine was delivered i.m. with EP using an Elgen Twinjector device [25] as described [24]. In each arm, the dose was escalated, with 5 patients per group: in arm I (without EP)—level 1: 800 μg, level 2: 1,600 μg, level 3: 3,200 μg per dose and in arm II (with EP)—level 1: 400 μg, level 2: 800 μg, level 3: 1,600 μg. If in the absence of safety concerns the immunological data supported this, patients were allowed to cross over between arms of the study after the first 3 vaccinations and receive the dose of the matched level in the opposite arm.

Follow-up on study was at weeks 0, 2 and 4 following vaccination, monthly to week 32 and then 2 monthly to week 72. At each visit, PSA levels were measured. For safety evaluation, full blood counts, clotting, serum biochemistry, LDH (lactate dehydrogenase), CK (creatine kinase) and autoimmune profiles of serum were monitored. PBMC were stored in liquid nitrogen for immunological assessment.

### Clinical follow-up

PSA values were available up to the date of consent, and during the 72-week study follow-up for the vaccinated cohort; ethical permission to collect time-point-matched PSA values for the unvaccinated patients was not obtained. PSA values used to calculate the PSA-DT were evaluated by a study-independent, blinded reviewer. Using only evaluable PSA values, PSA-DT was calculated using an algorithm calculator (http://mskcc.org/applications/nomograms/prostate/PsaDoublingTime.aspx). PSA-DT was calculated for each patient for the period pre-study, for 6-month periods on study and for the overall 72-week study period (calculated up to week 72 or until treatment). Time to next treatment and survival (assessed up to 31/12/2010) were recorded for all patients.

### Immunological evaluations

MIATA (Minimal Information About T-cell Assays; http://www.miataproject.org) guidelines were used to report immunological data on T-cell responses [26, 27] (Online Resource 1). PBMC were isolated from heparinized blood samples collected at each study visit. Recovery and viability were calculated using a manual hemocytometer and trypan blue exclusion. PBMC were cryopreserved and stored in LN_2_ vapor phase (Section 1, Online Resource 1). PBMC were assessed for immunological responses using assays validated to GCP for laboratories, and laboratory compliance was verified by external audit [28].

#### ELISPOT

PBMC from all follow-up time-points from each patient were assessed for IFNγ production in response to stimulation with recombinant FrC protein (20 μg/mL) [28] or PSMA_27_ (VLAGGFFLL) peptide (10 μg/mL, Protein Peptide Research, UK). The validated ELISPOT method used is described in detail in Section 2, Online Resource 1.

#### Cultured ELISPOT

PSMA_27_-specific CD8^+^ T cells were cultured in vitro for 8 days. As cell number was limiting, samples from different time-points were pooled and cultured in the following groups: baseline, weeks 8, 10 and 12, weeks 16, 20 and 24, weeks 26, 28 and 32 and weeks 50, 52 and 60. Cells were cultured with 10 μg/mL PSMA_27_ peptide or with a pool of viral peptides or a control peptide, HIV. IL-2 was added on days 3 and 6 and cells were harvested, washed and rested overnight on day 8. Following re-stimulation with 10 μg/mL peptide, IFNγ production was measured by ELISPOT. Full details are provided in Section 2, Online Resource 1.

### Statistical analysis

Median values are presented throughout, where appropriate with 25 and 75 % interquartile box with ranges. Significance was determined by either a two-sided, nonparametric Wilcoxon signed rank test or a Mann–Whitney test. A value of *p* < 0.05 was considered significant.

## Results

### Patient demographics

Sixty-four patients were eligible for the study (Fig. 1; Table 1). Thirty-two HLA-A2^+^ patients were vaccinated and 32 HLA-A2^−^ patients formed the control group for clinical follow-up. Two patients with adverse events (AE) after two vaccinations were replaced per study protocol and included in the safety but not in the immunological analyses (Fig. 1). One patient received three vaccinations before disease progression and commenced androgen suppression but remained evaluable for immune responses. After the initial 3 vaccinations with either DNA or DNA + EP, all but 4 patients went on to receive booster vaccines with EP (weeks 24 and 48). Twenty-nine patients completed vaccination and 72-week study follow-up (Fig. 1).

**Table 1:** Patient demographics for screened, eligible and consented patients

Parameter	Vaccinated	Non-vaccinated
	HLA-A2^+^	HLA-A2^−^
	*n* = 30 no. (%)	*n* = 32 no. (%)
Median age, years	71	75
Range	58–84	66–80
Prior treatment		
Prostatectomy	10 (30)	4 (13)
Radiation therapy	24 (80)	29 (91)
Androgen deprivation	25 (83)	27 (84)
Gleason score		
<6	15 (50)	10 (31)
7	14 (47)	16 (50)
>8	1 (3)	2 (6)
Unknown		4 (13)
Tumor size		
Small (T1c–T3a)	19 (63)	23 (72)
Large (T3b–T3c)	6 (20)	4 (13)
Unknown	5 (17)	5 (16)
Baseline PSA (ng/mL)		
Median	5.0	5.3
Range	0.5–26.3	0.97–48.0
<2.0	1 (3)	1 (3)
2.0–5.0	17 (53)	14 (44)
5.0–10.0	8 (27)	8 (25)
>10	4 (13)	9 (28)

### Safety and adverse events

Safety evaluation in the first two dose groups has been reported [24]. Full data for all patients on the study and a summary of AEs recorded are listed in Online Resource 2. The vaccine was safe and well tolerated. Most AEs were grade 1 or 2 and ranged from injection site reactions to flu-like symptoms, back pain and nail changes. Vaccination was discontinued due to AEs in two patients: one experienced grade 3 worsening of a pre-existing psoriasis, with causality assessed as likely vaccine related, and a second grade 3 AE was identified as worsening of pre-existing Parkinson’s disease, assessed as unlikely to be vaccine related. Two serious AEs were observed: one patient developed grade 2 peripheral edema and a second was admitted for a TURP. Both events resolved fully and the patients continued on study with no recurrence.

Previously, we reported the safety of EP in dose groups 1 and 2 by the measurement of muscle damage markers CK and LDH [24]. Patients in dose group 3 showed no increase (>twofold baseline) in either CK or LDH, and any increase observed at days 1 and 5 after vaccination returned to baseline level by day 14 (data not shown).

### Clinical outcome

**Fig. 2:**
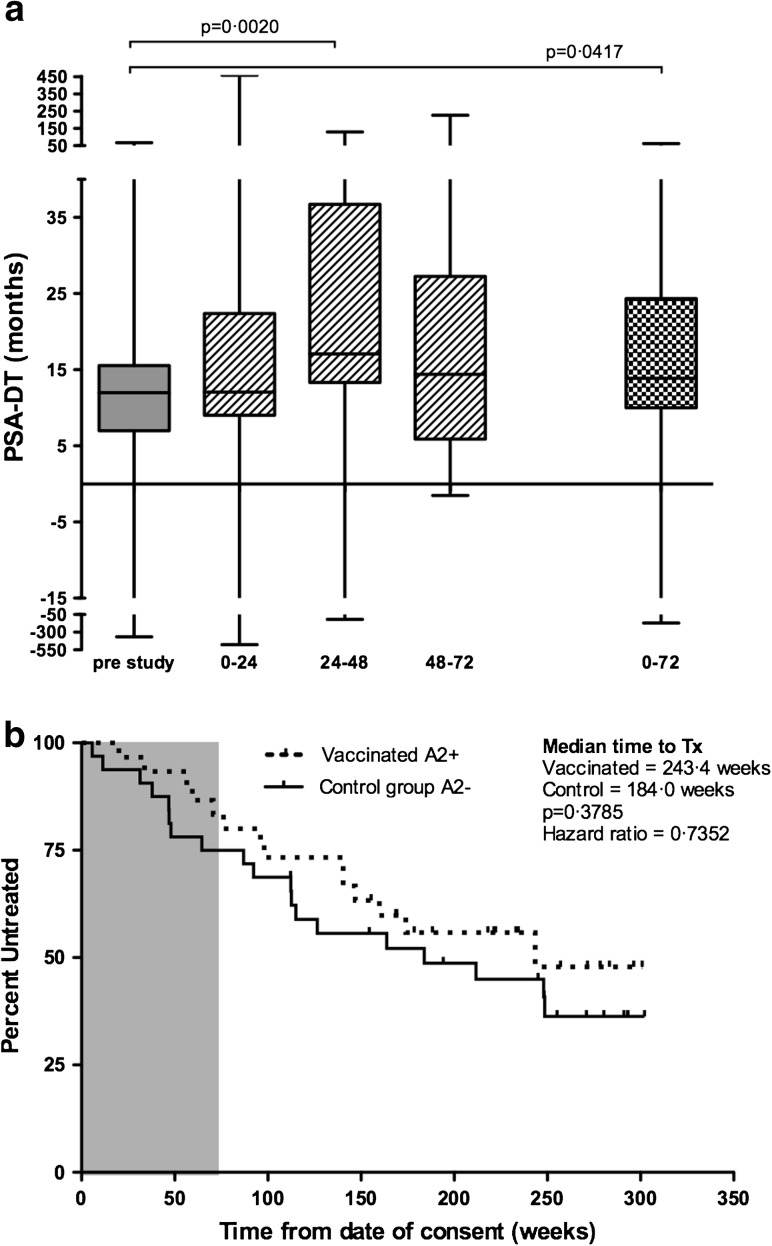
Clinical responses. **a** Shows a *box* and *whiskers plot* of PSA-DT calculated for each patient pre-treatment, for 6-month periods on study and overall for the whole 72-week study follow-up. Data represent the median and range for all HLA-A2^+^, vaccinated patients (*n* = 30). PSA-DT at weeks 24–48 and over the 72-week follow-up period is significantly increased over pre-treatment (*p* = 0.0020 and *p* = 0.0417, respectively). **b** A Kaplan–Meier plot of the time to next treatment. *Gray shading* indicates the on-study period. The small *vertical tick marks* show censored times. The *dashed line* represents vaccinated HLA-A2^+^ patients (*n* = 30), and *solid line* shows the unvaccinated HLA-A2^−^ control group (*n* = 32)

PSA-DT is widely used as an indicator of outcome [29] and was evaluated for vaccinated patients. PSA-DT showed significant increases during the study period (Fig. 2a), with an increase from 11.98 months (range −356.6 to 67.9) pre-study to 17.26 months (range −117.4 to 129.4) for the 24- to 48-month period post-vaccination (*p* = 0.0020 (Fig 2a)). This was a slow increase, not evident at the 0- to 24-month period. Over the whole study period (0–72 months), PSA-DT showed a significant increase (*p* = 0.0417) to 16.82 months (range −169.2 to 62.38), indicating a slowing of disease progression. Individual patient data are provided in Online Resource 3. Compared to baseline, PSA-DT increased in 24/30 patients at one or more time-points during the study and in 19/30 the PSA-DT increase continued to week 72. An increase of ≥200 % in PSA-DT at any point during the study was observed in 14/24, with 4/24 patients retaining this effect out to 72 weeks. Figure 2b shows time to next treatment in vaccinated patients compared to the control group. With a hazard ratio of 0.7352 (95 % confidence interval 0.37–1.45), time to next treatment was 243.3 weeks in the vaccinated group and 184.0 weeks in the control group (*p* = 0.3785).

There was no objective reduction in PSA. At a median of 4.6 years’ follow-up, 5 vaccinated patients compared to 6 control group patients have died. No effect of DNA dose on outcome was detected.

### Immunological responses

**Fig. 3:**
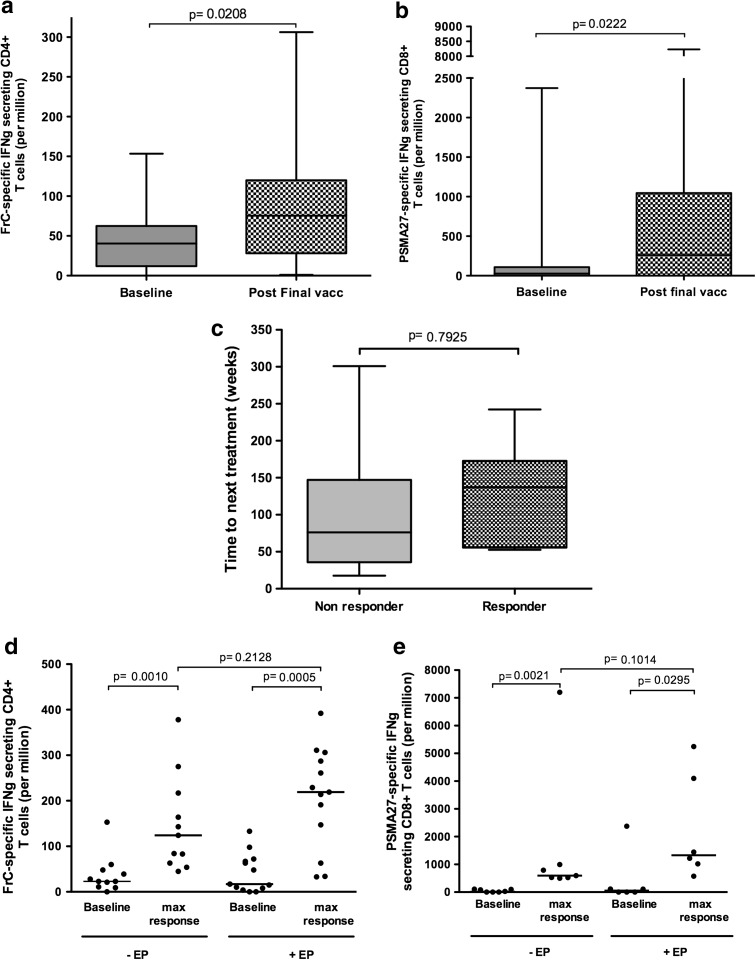
Immune responses. **a** and **b** Represent data from all patients who completed vaccination (*n* = 29) displayed as a *box* and *whiskers plot* and show the median and range of CD4^+^ and CD8^+^ IFNγ responses to FrC protein and PSMA_27_ peptide at baseline (*solid*) and post-final vaccination (checked). **c** Shows the time to next treatment for 14 patients that required additional treatment and compares patients who made a PSMA_27_-specific CD8^+^ response (responder, *n* = 7) with those that did not have a detectable response (non-responder, *n* = 7). **d** and **e**, *Scatter plots*, represent patients who made a significant response up to week 24, comparing patients receiving vaccination without (−EP) or with (+EP) EP. **d** Compares CD4^+^ responses to FrC at baseline and at week of max response (*n* = 11 and *n* = 13 for −EP and +EP, respectively). **e** Compares CD8^+^ responses to PSMA_27_ at baseline (*solid*) and at week of max response (*n* = 7 and *n* = 6 for −EP and +EP, respectively). All CD8^+^ responses have been assessed after short term in vitro culture

Immune monitoring was carried out on all patients, and responses are shown in Table 2. FrC-specific CD4^+^ and PSMA_27_-specific CD8^+^ T cells were evaluated using IFNγ ELISPOT. Ex vivo FrC-specific responses were used to assess vaccine operation and the effect of delivery modality. Of 30 patients, 29 had a significant CD4^+^ response to FrC following vaccination, in keeping with an expansion of a memory T-cell population detectable in the baseline samples. The median CD4^+^ T-cell response more than doubled by week 72 compared to baseline; baseline median IFNγ response was 34 spots/million (range 0–153) increasing to a median of 72 spots/million (range 1–306) at week 72 (*p* = 0.0208) (Fig. 3a).

**Table 2:** Summary of immune responses

Patient	ARM	Dose	Ab			CD4				CD8			CD8 (cultured)			
			±	Max Fold Inc.	Week of max.	±Week 0–24	±Wk 0–72	Max Fold Inc.	Week of max.	±	Max Fold Inc.	Week of max.	±Wk 0–24	± Wk 0–72	Max Fold Inc.	Week of max.
2	DDDDE	1	−			+	+	9.6	4	−			+	+	792	8–12
11	DDDDD	2	−			++	++	2.7	6	−			++	++	2,192	26–32
13	DDDDD	2	−			−	+	3.7	60	−			+	++	9.9	50–60
19	DDDDE	2	−			−	(+)	1.7	26	(+)	7	52	−	−		
1	DDDEE	1	++	73.9	50	++	++	8	60	−			−	−		
5	DDDEE	1	++	19.1	52	+	++	11	72	−			++	++	12.5	50–60
7	DDDEE	1	++	2.8	60	++	++	6	6	−			−	++	473	50–60
8	DDDEE	1	++	48.5	52	++	++	205	50	−			+	++	82.3	50–60
15	DDDEE	2	++	3.6	10	++	++	2.9	10	−			−	−		
17	DDDEE	2	−			+	+	2.5	10	++	201	26	+	+	993	8–12
23	DDDEE	3	++	11.3	52	+	++	2.5	50	−			−	−		
25	DDDEE	3	++	47.0	28	−	++	2.7	28	−			+	+	22.3	8–12
27	DDDEE	3	++	21.1	60	++	++	3.9	26	−			−	−		
29	DDDEE	3	−			−	++	2.4	72	−			−	−		
31	DDDEE	3	++	15.9	60	++	++	6	72	−			−	−		
3	EEEEE	1	++	2.9	12	++	++	63	12	++	72	48	−	−		
4	EEEEE	1	++	263	52	++	++	22	52	+	27	52	++	++	29	26–32
6	EEEEE	1	++	58.2	12	++	++	24	60	−			(+)	(+)	1.7	8–12
9	EEEEE	1	++	7.3	28	++	++	3.5	4	++	76	4	−	−		
10	EEEEE	1	++	42.8	12	−	−			−			−	−		
12	EEEEE	2	++	92.4	52	++	++	5.4	48	(+)	46	16	−	+	4.1	26–32
14	EEEEE	2	−			−	+	4.4	50	−			++	++	1,113	50–60
16	EEEEE	2	++	13.2	28	++	++	55	6	−			++	++	1,992	50–60
18	EEEEE	2	++	11.5	20	++	++	29	10	−			++	++	5,247	16–24
20	EEEEE	2	++	15.2	50	++	++	2	10	−			+	++	17	26–32
22	EEEEE	3	++	2.4	60	++	++	4.2	52	−			−	−		
26	EEEEE	3	−			++	++	4.6	16	−			−	−		
28	EEEEE	3	−			++	++	33	2	−			−	−		
30	EEE	3	+	2.1	16	+	+	3.4	12	−			−	−		
32	EEEEE	3	++	393	50	++	++	4	10	−			−	+	10.4	26–32

Data show whether the response is negative (−) (not significant), weakly positive (+) (<twofold increase but significant), positive (+) (>twofold increase and significant in one time-point) or strongly positive (++) (>twofold increase and significant in more than one time-point). If positive, the fold increase at the week of maximum response is shown

T-cell responses against PSMA_27_ were assessed in circulating lymphocytes. Effector CD8^+^ T cells are unlikely to persist in blood, and as expected, we found only low levels ex vivo (6/30 positive responses detected, 3/6 being observed at 2 or more time-points). To detect central memory cells, we cultured blood T cells with peptide/IL-2 for 8 days in vitro [30]; IFNγ-producing PSMA_27_-specific T cells were detected in 55 % (16/30) of patients. The median CD8^+^ response had increased 9.6-fold by week 72 compared to baseline (from 27 spots/million (range 0–2,373) to 260 spots/million (range 0–8,233) (*p* = 0.0222)) (Fig. 3b). There was a trend for patients with detectable PSMA_27_-specific T cells to have an increased time to next treatment (Fig. 3c, *p* = 0.7925); we identified no link between PSA-DT and detection of circulating peptide-specific CD8^+^ T cells. There was no apparent effect of DNA dose on immunogenicity.

### Effect of delivery on immune responses

The effect of vaccine delivery can be interpreted for the first 24 weeks, during which the 2 arms of the study remained independent. Thereafter, 11/15 patients crossed over to vaccination with EP for 2 doses per protocol based on an improvement in antibody responses to DOM with EP [24]. Figures 3d, e show CD4^+^ and CD8^+^ peak T-cell responses up to week 24, respectively. For both CD4^+^ and CD8^+^ responses, the delivery of vaccine ± EP generated a significant response compared to baseline. The effect of adding EP during delivery was not dramatic but there was a trend toward induction of higher levels of both CD4^+^ and CD8^+^ T-cell responses (*p* = 0.2128 for CD4^+^ T cells and *p* = 0.1014 for CD8^+^ T cells) (Fig. 3d, e). Clearly, larger numbers are required but this weak effect contrasts with the significant increase in humoral anti-DOM responses by adding EP [24].

## Discussion

In HLA-A2 transgenic mice, pDOM-PSMA_27_ epitope vaccination stimulates strong peptide-specific CD8^+^ T-cell responses [22]. The PSMA_27_ epitope is processed from PSMA, and induced T cells can kill human target cells, confirming PSMA_27_ as a useful target for CD8^+^ T-cell attack. The phase I/II study we present here takes these observations to the clinic. In HLA-A2^+^ prostate cancer patients at biochemical failure, with low disease burden, vaccination significantly increased PSA-DT compared to pre-vaccination. We compared time to next treatment in vaccinated patients with a synchronous group of HLA-A2^−^ patients. The data suggest that pDOM-PSMA_27_ vaccination could affect the natural history of prostate cancer and the suggestion that time to next treatment can be extended will need evaluation in a larger, randomized study. Whether HLA-A2 in its own right is an adverse prognostic factor has not been answered definitively, though there is a suggestion of link to prostate cancer incidence [31], increased proportion of large tumors (T3b–T3c) and higher post-operative Gleason sums compared to the HLA-A2^−^ control group [32]. An adverse effect of HLA-A2 on outcome would strengthen a clinical effect of vaccination.

The increase in PSA-DT became visible after >24 weeks after first vaccination, and in 14/30 patients, the increase was 200 % or greater. From a baseline of 12 months, PSA-DT increased to 17 months. While caution is needed in the absence of randomized controls [33], a consistent story supporting an effect of vaccination at biochemical recurrence is emerging, where vaccination significantly increases PSA-DT [6, 9, 34–36]. Within the limits of comparability between studies, it appears that our DNA vaccine, targeting a single PSMA epitope, is at least as effective as other more complex DNA- or peptide-based vaccines.

T cells against the DOM helper sequence expanded in almost all (29/30, 97 %) patients, demonstrating patients’ immunocompetence and the immunological performance of the vaccine. pDOM-PSMA_27_ induced CD8^+^ T-cell responses in 16/30 (55 %) of patients, using pre-defined assay criteria and a single round of in vitro culture. Comparison of immunogenicity between trials is hampered by widely varying assay systems used for immune monitoring, and additionally, only few studies are available that report this data in comparable clinical settings [6, 9]. The dataset by McNeel et al. [9] with a full-length DNA vaccine encoding PAP is most similar to our own, and in this study, 3/22 patients had measurable CD8^+^ T-cell responses compared to 6/30 patients in our dataset ex vivo.

Incorporation of full-length antigen sequence into the DNA vaccine seems attractive since it would allow vaccination of all rather than to the 40 % of patients who carry HLA-A2 [9]. However, there are cogent reasons for using a peptide-focused vaccine since the inductive power of the repositioned peptide is generally considerably higher than from full-length sequence [37]. CD8^+^ T cells specific for a single epitope are clearly capable of suppressing even an acute viral infection [38]. Should escape from focused attack occur, a second vaccine against a different epitope could be used [39], and we are exploring double attack in our current clinical trial against the WT-1 antigen [40]. Although our vaccine design could readily incorporate tumor-derived MHC class II-binding epitopes, there is no clear evidence that these are required for the maintenance of cytotoxic T cells and there is a danger that regulatory T cells might be induced [13, 41].

Viral vector–based vaccines have the problem of pre-existing or induced antiviral immunity. However, an MVA-MUC-1 vaccine induced an IFNγ^+^ T-cell response to MUC-1 after short-term culture in 7/34 patients with prostate cancer [6]. Pox viral delivery in metastatic disease also generated PSA peptide-specific CD8^+^ T-cell responses in 13/29 patients following PSA-TRICOM vaccination [42] and in 9/24 patients following MVA-Trovax vaccination [43]. It appears that our approach has at least comparable immunogenicity. We would contend, however, that avoiding blocking immunity, likely to arise from MVA [44], will be important for repeated vaccinations required to maintain attack on cancer. A concern at the outset of our study had been whether T-cell responses would be durable, as with some vaccines approaches CD8^+^ T-cell responses can be lost rapidly and then not re-expand after repeated injection [18]. Our data argue that with DNA vaccination this is not a problem with T-cell responses maintained to the end of the follow-up period.

To examine whether our DNA vaccine had sufficient potency to be scaled from mouse to human, we examined the delivery of our DNA vaccine using the Inovio Elgen100 device for the first time in the clinic. We had found pre-clinically [22, 45] that EP increased antibody responses, with lesser increase in CD8^+^ T-cell responses to our DNA fusion vaccine. In the clinic, this dichotomy is also evident with clear increases in antibody [24] but only a trend for increase in both CD4^+^ and CD8^+^ T-cell responses with EP. After cross-over of 11/15 patients to EP boosting, there is a significant and durable increase to the end of the study but we can no longer assess the impact of the individual delivery modalities. It is intriguing to speculate why EP has an apparently smaller effect on T-cell responses compared to humoral responses. In the trial, this may simply be a reflection of very small patient numbers treated without electroporation, and a randomized dataset needs to evaluate the comparative question further. A possible explanation for both the murine and human data could be that unlike B-cell responses, where the increased muscular antigen expression after electroporation leads to higher humoral responses [24], for T cells there may not be such a strict correlation with the quantity of antigen expressed by the muscle cells.

In summary, the pDOM-PSMA_27_ vaccine is safe, generates anti-PSMA responses in the majority of patients and is associated with an increase in PSA-DT. Use of EP was well tolerated and may increase T-cellular vaccine efficacy. These findings merit further testing in a randomized setting. Examining the vaccine-induced T cells for their ability to home to the tumor will be a critical component of further evaluation and may offer the tool to better identify a link between vaccine-induced immunity and clinical outcome.

## Electronic supplementary material

Below is the link to the electronic supplementary material.

Supplementary material 1 (PDF 235 kb)
