# Supplementary material for: DNA fusion-gene vaccination in patients with prostate cancer induces high-frequency CD8+ T-cell responses and increases PSA doubling time
Source: Cancer Immunol Immunother. 2012 May 22;61(11):2161–70. doi: 10.1007/s00262-012-1270-0 (PMC3493666; doi:10.1007/s00262-012-1270-0)
Supplement: Supplementary file 1 — Supplementary material 1 (PDF 235 kb) [file 262_2012_1270_MOESM1_ESM.pdf]

Online Resource 1: MIATA reporting of T-cell assays

MODULE 1: INFORMATION ON THE SAMPLE

IA: DONOR

Patient demographics can be found in Table 1.

1B: SOURCE

Blood was collected in Lithium/Heparin blood tubes at each study visit for each of the vaccinated patients. The time between drawing and processing was <4hrs. Samples taken at Royal Marsden Hospital were couriered as soon as possible after drawing and transferred at room temperature to Southampton for processing.

PBMC were purified by density gradient centrifugation using Lymphoprep (Axis Shield). PBMC were counted and viability checked prior to freezing using manual cytometer and Trypan Blue.

1C: CRYOPORESERVATION AND STORAGE

All assays were carried out on cryopreserved PBMC. PBMC were frozen at a concentration of 5-10 x10^6 per vial in 50% human AB serum (Sigma batch tested) 40% RPMI 1640 (+ sodium pyruvate and PSG) and 10% DMSO. Freezing additives were added at room temperature before aliquotting cells into cryovials. The vials were then transferred to a Nalgene Mr Frosty and kept at -80ºC overnight. Vials were then stored in Liquid Nitrogen until the full time course had been collected.

PBMC were stored in Liquid Nitrogen for a range of 20-38 months before thawing and testing (based on baseline samples, which were stored for the longest period for each donor).

1D: QUALITY OF CELL MATERIAL

Mean viability after thawing was 94% (range 70-100). Mean recovery of PBMC after thawing was 89% (range 41-123%), as determined by a % percentage of cell input number before freezing and counting by manual haemocytometer and Trypan Blue post thawing respectively.

MODULE 2: INFORMATION ON THE ASSAY

2A: CELL COUNTING

Mean viability after thawing was 94% (range 70-100). Mean recovery of PBMC after thawing was 89% (range 41-123%), expressed as a percentage of cell input number before freezing and counting by manual haemocytometer and Trypan Blue.

2B: MEDIUM/SERUM

Ex vivo CD8 and CD4 ELISPOT assay:
RPMI 1640 + sodium pyruvate + PSG+ 10% Human AB Serum (Sigma batch tested)

Cultured ELISPOT assay:
Xvivo 15 + 5% human AB serum (Lonza 14-498E)

2C: THE ASSAY

Ex vivo CD8 and CD4 ELISPOT:
Clear Multiscreen 96-well ELISPOT plates (Millipore, MAIPS4510) were pre-coated with 15 µg/ml anti-human IFNγ antibody (mAb 1 D1 K, Mabtech), and left overnight at 4°C. Thawed PBMCs (4 x10^5/well) were incubated in triplicate for 20 h (CD8+) or 40 h (CD4+), at 37°C, 5% CO_2 with medium only, test antigen (CD8+: 10 µg/ml peptide (PSMA27, 27–35, (VLAGGFFL) ,a pool of viral peptides comprising equal amounts of cytomegalovirus (CMV), CMV pp65 493–499, (NLVPMVAVT); influenza A, Matrix 1 58–66, (GILCFVFTL); Epstein Barr virus (EBV), BMLFI 259–217 (GLCTLVAML), and measles, non-structural C protein 84–92 (KLWESPQEI) [16]. An HIV peptide, IV9 RT 476–484 (ILKEPVHGV) [17] served as negative control). HLA A0201-binding peptides were obtained, with certificates of 95% purity, from Peptide Protein Research Ltd (Fareham, UK). (CD4+: 20 µg/ml recombinant FrC protein. Recombinant c kappa-tagged protein was generated in house, using a mammalian 293-F expression system (Freestyle, Invitrogen), and purified on a column of polyclonal sheep anti-human free kappa linked to Sepharose 4B beads. Protein quality was confirmed on SDS gels and anti-kappa blots and endotoxin levels were tested before use in ELISPOT. In both assays 5 µg/ml Phytohemagglutinin (PHA) was used to confirm that cells were able to produce detectable IFNγ. IFNγ secreting memory T-cells specific for the antigen were detected as spots using 1 µg/ml biotinylated IFNγ antibody (mAb 7B61 biotin, Mabtech) followed by 1 µg/ml streptavidin alkaline phosphatise (Mabtech) and a BCIP/ NBT detection kit (Zymed).

Cultured ELISPOT:
Cells were cultured in 2mL Xvivo 15 media (Invitrogen) + 5% human AB serum (Lonza, batch tested) at a concentration of 2 x10^6 per well (COSTAR, 12 well culture plate) with 10µg/mL of peptide. Cells were either cultured with PSMA27, (VLAGGFFL) or, as a positive control, a pool of viral peptides (as above). HIV peptide (ILKEPVHGV) served as negative control. The plates were incubated at 37ºC 5% CO_2. On days 3 and 6, 1mL of media was removed from each well and replaced with 1mL 20IU/ml IL-2 (R&D systems). On day 8 the cells were harvested, washed 3 times and rested overnight in CRPMI +10% AB serum at 37C, 5% CO_2. The following day the cells were counted and added to ELISPOT plates previously coated with anti-IFNγ antibody at a concentration of 50,000 cells per well (or 5,000 cells per well for cells stimulated in culture with viral antigens). The cells were stimulated in the plate overnight with 10µg/mL of peptide matched to the peptide they were stimulated with in culture, HIV peptide or with CRPMI +10% AB serum with no peptide. Spots were developed using method above for ex vivo ELISPOT.

2D: CONTROLS

Ex vivo CD8 and CD4 ELISPOT assay:
A negative control of media only and HIV peptide (CD8 only) and positive control of PHA were used.

Cultured ELISPOT assay:
A pool of viral peptides was used as a positive control for the in vitro culture. In the ELISPOT, cells were tested against media only and HIV peptide as a negative control.

MODULE 3: DATA ACQUISITION

3A: EQUIPMENT AND SOFTWARE

ELISPOT plates were read on an AID ELISPOT plate reader (Autoimmun diagnostika GmbH) using software ELR04.

3B: ACQUISITION STRATEGY

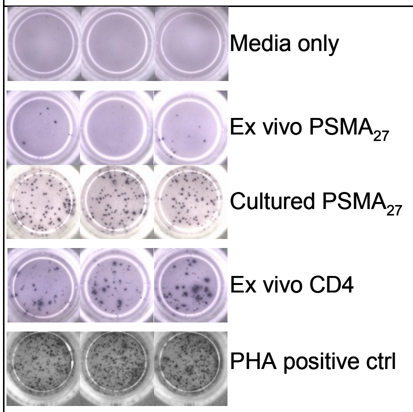

Example of raw data: representative wells taken from Ex vivo CD8, Ex vivo CD4 and cultured ELISPOT assays

MODULE 4: THE INTERPREATION OF RESULTS

4A: RAW DATA

Plates were read and data calculated as follows:

The spots per well were recalculated to spots per million PBMC.
The mean of media control triplicate subtracted from antigen specific samples.
Mean and SD were then calculated for each triplicate for antigen specific responses.

Below is a table of median (and range) spots per million PBMC from every patient and time point measured following stimulation with media only or antigen (PSMA27 or FrC).

	**All samples**		**Baseline**		**Weeks 2-72**	
	**Median**	**Range**	**Median**	**Range**	**Median**	**Range**
**CD8 ELISPOT**						
Ex vivo background (media only)	8	0-242	10	0-234	8	0-242
Antigen-specific response (PSMA27)	3	0-201	2	0-29	3	0-201
**CD4 ELISPOT**						
Ex vivo background (media only)	8	0-220	15	0-87	8	0-220
Antigen-specific response (FrC)	60	0-396	39	0-153	63	0-396
**CULTURED ELISPOT**						
Cultured background (media only)	188	0-3075	207	0-1147	187	0-3075
Antigen-specific response (PSMA27)	148	0-8233	27	0-2373	266	0-8233

Raw data may be provided upon request.

4B: RESPONSE DETERMINATION, STATISTICAL TESTS AND EMPIRICAL RULES

A positive response in the ELISPOT was determined by:

a) more than 25 spots per million PBMC

b) the antigen specific response must be 2xSD above the media control

c) for a response between time points, the time point must be 2xSD above the baseline antigen specific response, 2 fold increase above baseline and significant when tested by Student T Test.

A negative response - is a patient with no time points that meet the above criteria.

A weak positive (+) is a patient whose response is significant by T test but is less than two fold above baseline.

A positive responder + has one time point that meets the above criteria

A strong positive responder ++, has more than one time point that meets the above criteria

Definition criteria were pre-defined before the study commenced.

Data exclusion applied on a maximum of three wells per plate; For example, an outlier could be removed from a triplicate.

MODULE 5: THE LABORATORY ENVIRONMENT

5A: GENERAL LABORATORY OPERATION

These studies were conducted in a laboratory that operates to GCLP.

5B: LABORATORY PROCEDURE STANDARDIZASTION

These studies were performed using standard operating protocols covering all aspects from processing of cells, the assay, the data acquisition and data interpretation.

5C: STATUS OF ASSAY QUALIFICATION AND VALIDATION

These studies were performed using validated assays.

Online Resource 2: Adverse Events believed to be likely or possibly related to vaccination

Adverse Event	Grade 1 No. (%)	Grade 2/3 No. (%)
**Injection site reaction:**		
Haematoma, Pain, Bruising, Oedema, Induration	5 (16)	2 (6)
**Constitutional symptoms:**		
Flu-like syndrome	2 (6)	
**Musculoskeletal**		
Back pain		1 (3)
**Other**		
Psoriasis*		1 (3)
Nail changes	1 (3)	
TURP		1 (3)
Parkinson's diease*†		1 (3)

Data represent the number of patients (out of 32) that reported an event at any time during the 72 week follow-up period, with the highest grade being recorded if more than one event was reported for one patient. *These patients discontinued vaccination and were replaced on study. †Deemed unlikely to be caused by the vaccine.

Online Resource 3: PSA Doubling Times

PATIENT	PSA Doubling Time in Months (No. Months, No. PSA Values Used for Calculation)				
	Pre-treatment	Weeks 0-24	Weeks 24-48	Weeks 48-72	Weeks 0-72
1	13·6 (27,8)	11·9 (6,10)	**35·3** (6,6)	**227·4** (7,4)	13·7 (18,21)
2	5·7 (10,7)	9·0 (6,10)	**12·6** (6,6)	3·1 (1, 2 *)	8·7 (13,18*)
3	7·7 (38,9)	6·2 (6,10)	**16·6** (6,6)	9·5 (6,4)	10·8 (18,20)
4	31·7 (55,12)	**115·7** (6,10)	42·0 (6,6)	17·5 (6,4)	48·3 (18,20)
5	-51·8 (9,8)	9·6 (6,10)	54 6,8)	20·8 (5,6)	18·0 (17,24)
6	8·7 (7,5)	9·1 (6,10)	6·9 (6,6)	3·8 (5,4)	7·1 (17,20)
7	67·9 (18,6)	65·2 (6,9)	**-117·4** (6,7)	32·5 (5,7)	58·7 (17,23)
8	16·5 (47,12)	20·7 (6,9)	16·4 (6,6)	**39·8** (6,6)	25·5 (18,21)
9	11·8 (53,17)	17·0 (6,10)	14·7 (6,6)	5·1 (6,3)	9·2 (18,19)
10	30·4 (36,7)	24·5 (6,10)	14·9 (6,6)	24·0 (6,5)	**62·6** (18,21)
11	15·2 (23,7)	9·0 (5,10)	27·5 (5,8)	9·9 (6,7)	17·3 (16,25)
12	6·3 (7,6)	**21·6** (6,6)	**17·0** (6,6)	4·6 (1,2 *)	13·5 (13,14*)
13	10·8 (25,7)	**224·3** (6,10)	17·4 (5,7)	18·1 (6,7)	**23·94** (17,24)
14	-57·3 (9,5)	8·4 (6,9)	129·4 (6,6)	16 (5,4)	16·8 (17,19)
15	6·9 (2,4)	11·9 (6,10)	5·8 (6,9*)	-	9·62 (12,19*)
16	12·2 (27, 9)	7·1 (5,10)	3·5 (6,7)	1·75 (5,6*)	4·5 (16,23*)
17	14·0 (10,6)	12·1 (5,10)	22·4 (6,8)	**128·0** (5,7)	18·1 (16,25)
18	59·6 (24,8)	14·4 (5,10)	65·5 (7,8)	26·6 (6,7)	27·9 (18,25)
19	10·3 (52,13)	11·8 (5,11)	14 (6,7)	5·0 (5,5)	11·7 (16,23)
20	10·5 (9,7)	8·9 (6,10)	14·2 (6,6)	12·6 (6,4)	11·1 (18,20)
22	28·2 (30,5)	**459·8** (6,10)	**63·9** (5,7)	28·5 (6,7)	**-169·2** (17,24)
23	12·2 (51,8)	**-476·2** (7,10)	**38·0** (5,8)	**27·4** (5,7)	**25·5** (17,25)
25	12·1 (11,5)	**31·6** (6,10)	11·7 (6,6)	12·3 (6,3)	13·5 (18,19)
26	12·5 (52,9)	19·1 (6,10)	24·5 (6,8)	8·0 (6,7)	14·0 (18,25)
27	20·1 (56,7)	**50·1** (5,9)	**45·0** (4,5)	12·8 (11,4)	34·4 (20,18)
28	-7·1 (10,6)	13·3 (5,10)	11·9 (6,6)	112·7 (6,4)	18·2 (17,20)
29	7·0 (14,6)	7·3 (6,10)	**33·9** (6,8)	10·2 (6,8)	10·3 (18,26)
30	7·6 (9,7)	2·4 (4,8 *)	-	-	2·4 (4,8*)
31	14·7 (11,6)	9·7 (6,10)	14·0 (6,8)	18·8 (6,5)	10·1 (18,23)
32	-365·6 (2,4)	16·5 (6,10)	18·1 (6,7)	-1·51 (5,3)	20·2 (17,20)
**Median**	11·98	12·15	17·26	17·56	16·82
**Range**	-356·6 to 67·9	-476·2 to 459·8	-117·4 to 129·4	-1·5 to 227·4	-169·2 to 62·6

PSA-DTs were calculated for each patient pre-treatment, for 6 monthly periods on study and for the whole study follow-up period (week 0-72). PSA values pre-treatment were evaluated by a blinded expert and only evaluable PSA's were used. The number of months and the number of values used to calculate the DT are in parentheses. Bolded PSA-DT indicates a >200% increase over pre-treatment. *Denotes patients received treatment and further PSA values were not included in PST-DT calculation.
